# Comparative microbiome analysis of parasitic and necrophagous subspecies of* Lucilia cuprina*

**DOI:** 10.1007/s00253-026-13969-w

**Published:** 2026-08-03

**Authors:** Shilpa Kapoor, Ying Ting Yang, Madison Bone, Aaron R. Jex, Simon Baxter, Andrew P. A. Oxley, Michelle L. Harvey, Philip Batterham, Robin B. Gasser, Vernon M. Bowles, Trent Perry, Clare A. Anstead

**Affiliations:** 1 Department of Veterinary Biosciences, Melbourne Veterinary School, Faculty of Science, The University of Melbourne, Parkville, VIC 3010 Australia; 2 Infection and Global Health, Walter and Eliza Hall Institute of Medical Research, Parkville, VIC 3052 Australia; 3 Bio21 Molecular Science and Biotechnology Institute, The University of Melbourne, Parkville, VIC 3010 Australia; 4 School of Life and Environmental Sciences, Deakin University, Geelong, VIC 3216 Australia

**Keywords:** Australian sheep blowfly, Flystrike, Bacteria, 16S rRNA sequencing

## Abstract

**Abstract:**

Blowflies of the genus *Lucilia* (Insecta: Diptera: Calliphoridae) are important in forensic science, medical research, and agriculture, particularly due to their role as facultative ectoparasites of domesticated sheep. The Australian sheep blowfly, *L. cuprina dorsalis*, is a primary causative agent of cutaneous myiasis, leading to substantial economic losses to the wool industry. In contrast, the closely related subspecies *L. cuprina cuprina* does not induce myiasis in sheep, but functions as a necrophagous subspecies in urban regions of Australia. Differences in the physiology, behavior, and host interactions of these subspecies might be attributed to their bacterial community structures as demonstrated in other blowfly species. However, our understanding of the bacterial communities associated with these flies is limited. This study aimed to characterize the microbiomes of two geographically distinct subspecies of *L. cuprina*—*L. c. dorsalis* (rural) and *L. c. cuprina* (urban) using 16S rRNA gene sequencing. The findings indicate that, while both subspecies harbor broadly similar microbial compositions, their respective urban and rural environments influence the prevalence of specific bacterial genera. The bacterial communities of these flies were predominantly composed of members of the phyla *Proteobacteria*,* Firmicutes*, *Bacteroidetes*, and *Actinobacteria*. The genus *Ignatzschineria* was highly abundant in *L. c. dorsalis*, whereas *Pseudomonas* was more prevalent in *L. c. cuprina*, highlighting distinct microbial compositions between the two subspecies. These findings provide fundamental insights into the microbial composition of *Lucilia* species, contributing to a broader understanding of their ecological and biological roles, with potential applications in forensic entomology, medical research, and agricultural pest management.

**Key points:**

• *The microbial communities of Lucilia cuprina dorsalis and Lucilia cuprina cuprina have a broadly similar composition, but the abundance of particular bacterial genera is notably different.*

• *Proteobacteria is the most abundant phylum in both subspecies.*

• *The genus Ignatzschineria is notably abundant in Lucilia cuprina dorsalis, while Pseudomonas is predominant in Lucilia cuprina cuprina, emphasizing differences between the microbial community members between these subspecies.*

**Graphical abstract:**

**Figure:**
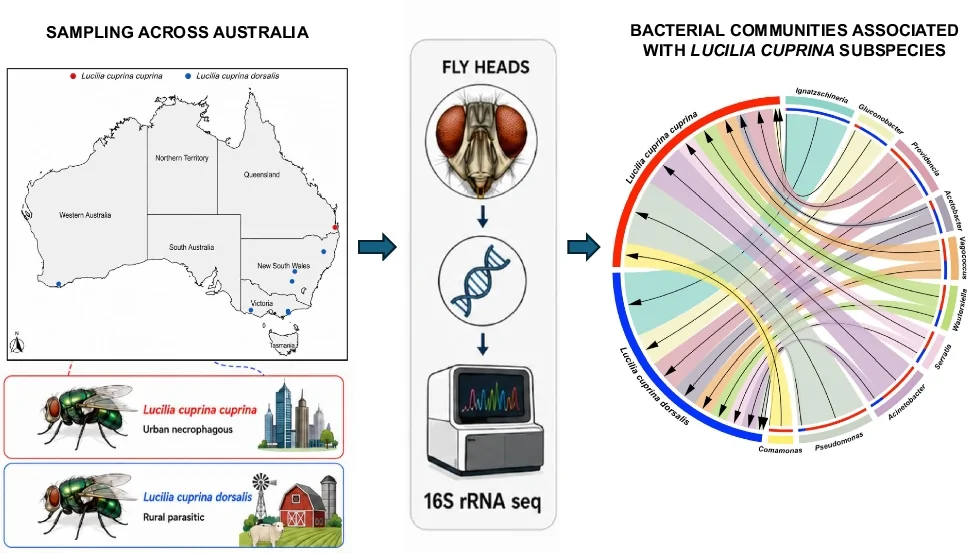

**Supplementary Information:**

The online version contains supplementary material available at https://doi.org/10.1007/s00253-026-13969-w.

## Introduction

Microbial associations with insects are well-documented (Hilker and Meiners 2008; Ma et al. 2012b; Schröder and Hilker 2008), with bacterial communities shown to be capable of influencing life-history traits (Ma et al. 2012a), sex ratios (Hurst and Jiggins 2000) and population structure (Denk-Lobnig and Wood 2025; Virgo et al. 2025). Previous research has established that blowflies, like many other insects, harbor diverse symbiotic microorganisms that influence their physiology, immunity and host-seeking behaviors in parasitic species (Gasz et al. 2021; Palevich et al. 2021; Singh et al. 2015; Weiss et al. 2011). For example, the bacterial genus *Ignatzschineria* has been identified in both larval and adult stages of some blowfly species, suggesting a potential role in modulating host behavior and attraction to carrion or wounds (Heddema et al. 2016; Iancu et al. 2020). Additionally, microbial metabolites have been shown to modulate insect attraction to hosts, as observed in the dipterans *Musca domestica* (Lam et al. 2007), *Aedes aegypti* (Ponnusamy et al. 2008) and the blowfly *L. sericata* (Ma et al. 2012b; Tomberlin et al. 2017). Given the demonstrated impact of bacterial associations on blowfly behavior, further microbiome research is likely to yield insights relevant to forensic entomology, medical applications, veterinary science, and agricultural pest management (Tomberlin et al. 2017).

Blowflies, particularly species within the genus *Lucilia* (Insecta: Diptera: Calliphoridae), are of major global ecological and economic significance (Anstead et al. 2015; Heath and Bishop 2006; Kapoor et al. 2025b). They function as primary decomposers in ecosystems (Galante and Marcos-Garcia 2008; Kasper 2013), serve as valuable tools in forensic entomology for the estimation of *post mortem* intervals (Stevens and Wall 2001; Vanin et al. 2008; Verma and Paul 2016), and are utilized in medical maggot therapy for wound debridement (Nezakati et al. 2020; Nigam et al. 2010; Paul et al. 2009). For example, *L. c. cuprina* (Wiedemann, 1830) is a necrophagic subspecies found primarily in urban areas. *L. c. cuprina* has been documented in Australia, China, Fiji, Hawaii, India, Indonesia, Japan, Java, Malaysia, the Americas, and Timor (Kapoor et al. 2025a, 2023; Nelson et al. 2012; Norris 1990; Stevens et al. 2002), and plays an important role in nutrient recycling and pollination (Cook et al. 2020; Kapoor et al. 2025a).

In addition to the positive roles they play, some *Lucilia* species are well known for their negative impact. For example, the common green bottle fly, *L. sericata* (Meigen, 1826), is the principal initiator of cutaneous myiasis, a debilitating condition in domesticated sheep in Europe but functions as a saprophagous species in various other regions around the world (Bisdorff and Wall 2008; Diakova et al. 2018; Kapoor et al. 2023; Wallman et al. 2005). In Australia, the Australian sheep blowfly, *L. cuprina dorsalis* (Robineau-Desvoidy, 1830), is a parasitic subspecies of *L. cuprina* (Wiedemann, 1830) and the primary causative agent of myiasis that results in significant economic loss (~ 324 M/annum) to the wool industry (Anstead et al. 2017; Shephard et al. 2022; Stevens and Wall 1997). *Lucilia cuprina dorsalis* is also distributed across Africa, Australia, India, and New Zealand (Bishop 1995; Kapoor et al. 2024, 2023; Nelson et al. 2012; Norris 1990), where it plays a similar parasitic role (Anstead et al. 2017; Heath and Bishop 2006; Kapoor et al. 2025b). These differences in physiology, behavior, and host interactions of *Lucilia* species and subspecies could be attributed to their bacterial community structures and influence their distribution in urban *versus* rural habitats. Yet, the bacterial diversity of *L. c. cuprina* remains largely unexplored. Previous research has only examined the microbiomes of *L. c. dorsalis* and *L. sericata* (Gasz et al. 2021; Iancu et al. 2021; Palevich et al. 2021; Singh et al. 2015).

This study aimed to characterize and compare the microbiomes of two geographically distinct *L. cuprina* subspecies—*L. c. dorsalis* (rural; parasitic) and *L. c. cuprina* (urban; necrophagous) in Australia using a 16S rRNA gene sequencing approach. This approach has the sensitivity to identify atypical bacterial groups or wild isolates at the genus level, even if they are not represented in current reference databases (Janda and Abbott 2007). This study provides the first comprehensive characterization of the microbiome of *L. c. cuprina* and the first comparative analysis with the microbiome of *L. c. dorsalis.*

## Materials and methods

### Sample collection

**Fig. 1:**
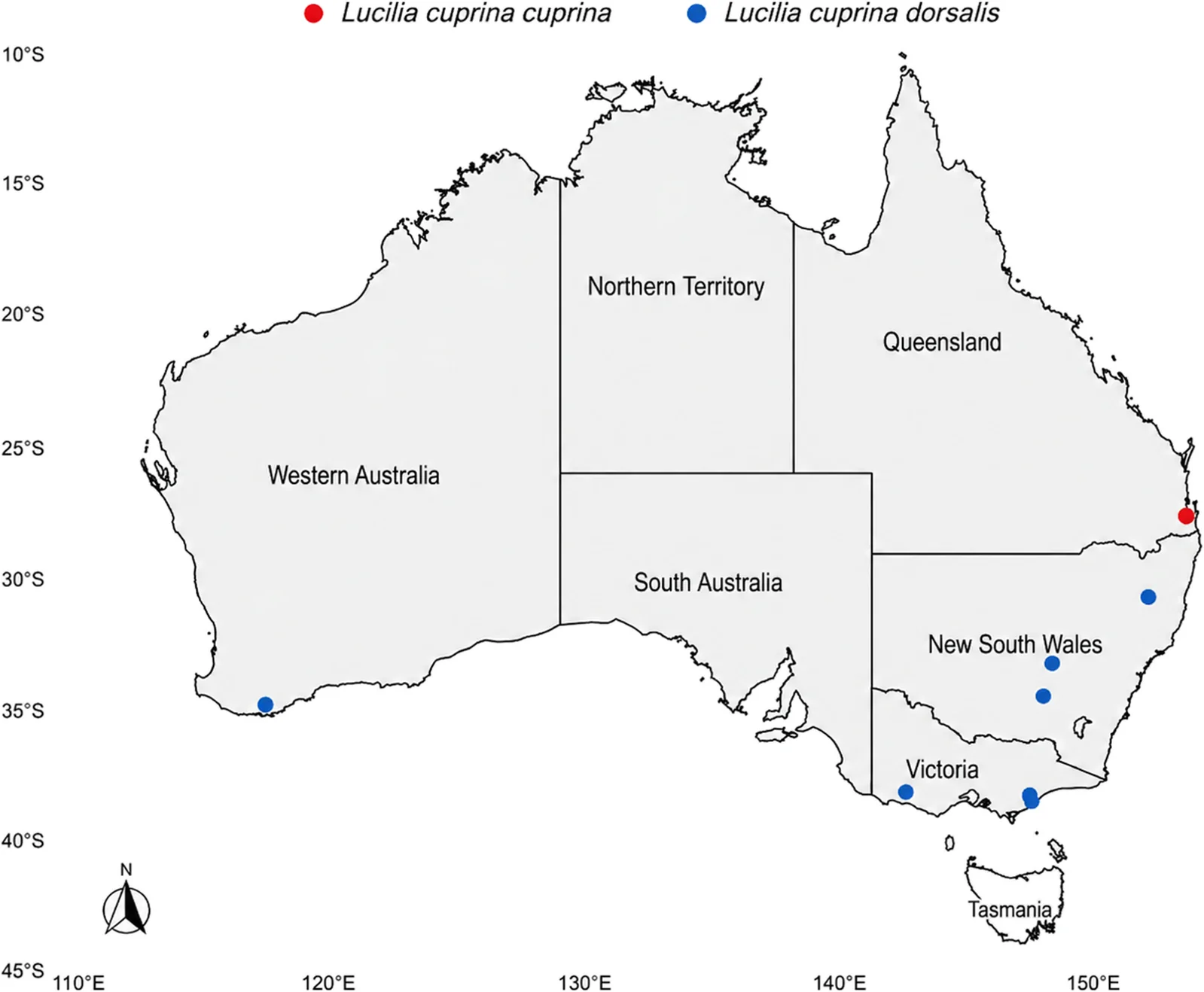
Map of Australia showing total *Lucilia cuprina* collection sites. Red circles indicate sampling locations for *Lucilia cuprina cuprina*, while blue circles represent collection sites for *Lucilia cuprina dorsalis*

Blowflies were trapped from four different states (New South Wales, Queensland, Victoria, and Western Australia) of Australia (February-April 2018) (Fig. 1; Table 1). Adults were trapped and preserved in a tissue stabilization and storage reagent (RNAlater) (Malmstrom 2015). The adult flies were identified as *L. c. dorsalis* or *L. c. cuprina* based on their morphological characteristics (Akbarzadeh et al. 2015; Holloway 1991; Marshall et al. 2011; Wallman et al. 2005; Waterhouse and Paramonovo 1950; Williams and Villet 2013) and were further genotyped for confirmation of the subspecies (Kapoor et al. 2025b).

**Table 1:** Geographical collection locations of the two *Lucilia cuprina* subspecies

Sample name	Collection site	Latitude	Longitude
*Lucilia cuprina dorsalis*			
BJN1C	Victoria	38.2763° S	147.0354° E
BJN2D	Victoria	38.2763° S	147.0354° E
BJN3C	Victoria	38.2763° S	147.0354° E
BJN3E	Victoria	38.2763° S	147.0354° E
BJN3F	Victoria	38.2763° S	147.0354° E
BJN4A	Victoria	38.2763° S	147.0354° E
BJN4F	Victoria	38.2763° S	147.0354° E
CA10	Victoria	37.8752° S	142.2901° E
CA12	Victoria	37.8752° S	142.2901° E
CA15	Victoria	37.8752° S	142.2901° E
CA17	Victoria	37.8752° S	142.2901° E
CA20	Victoria	37.8752° S	142.2901° E
CA22	Victoria	37.8752° S	142.2901° E
CA24	Victoria	37.8752° S	142.2901° E
CA3	Victoria	37.8752° S	142.2901° E
CA4	Victoria	37.8752° S	142.2901° E
CA5	Victoria	37.8752° S	142.2901° E
CA6	Victoria	37.8752° S	142.2901° E
CA8	Victoria	37.8752° S	142.2901° E
CA9	Victoria	37.8752° S	142.2901° E
JB10	Victoria	37.9661° S	147.0799° E
JB11	Victoria	37.9661° S	147.0799° E
JB12	Victoria	37.9661° S	147.0799° E
JB15	Victoria	37.9661° S	147.0799° E
JB17	Victoria	37.9661° S	147.0799° E
JB18	Victoria	37.9661° S	147.0799° E
JB19	Victoria	37.9661° S	147.0799° E
JB1B	Victoria	37.9661° S	147.0799° E
JB21	Victoria	37.9661° S	147.0799° E
JB24	Victoria	37.9661° S	147.0799° E
JB2B	Victoria	37.9661° S	147.0799° E
JB4	Victoria	37.9661° S	147.0799° E
JB5	Victoria	37.9661° S	147.0799° E
JB7	Victoria	37.9661° S	147.0799° E
JB9	Victoria	37.9661° S	147.0799° E
JG1	Western Australia	34.6253° S	117.6616° E
JG10	Western Australia	34.6253° S	117.6616° E
JG11	Western Australia	34.6253° S	117.6616° E
JG12	Western Australia	34.6253° S	117.6616° E
JG13	Western Australia	34.6253° S	117.6616° E
JG15	Western Australia	34.6253° S	117.6616° E
JG16	Western Australia	34.6253° S	117.6616° E
JG18	Western Australia	34.6253° S	117.6616° E
JG19	Western Australia	34.6253° S	117.6616° E
JG2	Western Australia	34.6253° S	117.6616° E
JG22	Western Australia	34.6253° S	117.6616° E
JG6	Western Australia	34.6253° S	117.6616° E
JG7	Western Australia	34.6253° S	117.6616° E
JG8	Western Australia	34.6253° S	117.6616° E
JG9	Western Australia	34.6253° S	117.6616° E
JSA	New South Wales	30.5036° S	151.6523° E
JSB	New South Wales	30.5036° S	151.6523° E
JSC	New South Wales	30.5036° S	151.6523° E
JSD	New South Wales	30.5036° S	151.6523° E
JSE	New South Wales	30.5036° S	151.6523° E
JSF	New South Wales	30.5036° S	151.6523° E
JSG	New South Wales	30.5036° S	151.6523° E
JSH	New South Wales	30.5036° S	151.6523° E
JSI	New South Wales	30.5036° S	151.6523° E
JSJ	New South Wales	30.5036° S	151.6523° E
JSK	New South Wales	30.5036° S	151.6523° E
JSO	New South Wales	30.5036° S	151.6523° E
JSP	New South Wales	30.5036° S	151.6523° E
JSQ	New South Wales	30.5036° S	151.6523° E
JSR	New South Wales	30.5036° S	151.6523° E
JSS	New South Wales	30.5036° S	151.6523° E
SP1	New South Wales	34.4468° S	147.5339° E
SP7	New South Wales	34.4468° S	147.5339° E
SR3	New South Wales	33.1200° S	147.9019° E
SR4	New South Wales	33.1200° S	147.9019° E
SR5	New South Wales	33.1200° S	147.9019° E
*Lucilia cuprina cuprina*			
GB1.2A	Queensland	27.4865° S	153.0360° E
GB10.1A	Queensland	27.4954° S	153.0252° E
GB16.1B	Queensland	27.4865° S	153.0360° E
GB9.1A	Queensland	27.5657° S	153.0572° E
GB9.1B	Queensland	27.5657° S	153.0572° E
GB9.1C	Queensland	27.5657° S	153.0572° E
GB9.1E	Queensland	27.5657° S	153.0572° E
GB9.1H	Queensland	27.5657° S	153.0572° E

### DNA extraction and sequencing

Genomic DNA was extracted from individual blowfly heads (*n* = 79) after rinsing with dH_2_O using CTAB/phenol chloroform (Green and Sambrook 2017; Stevens and Wall 1995), as the heads house the salivary glands, which are expected to harbor a high bacterial load. This organ has previously been identified as a bacterial reservoir, potentially playing a role in the persistence and transmission of bacteria within the blowfly (Singh et al. 2015). DNA quality was assessed using a 1% agarose gel in Tris–acetate-EDTA (TAE) buffer and DNA quantity was measured using a Qubit Fluorometer (Invitrogen). The extracted DNA was sent to the Australian Genome Research Facility Limited (AGRF) to be used as a template for 16S rRNA gene amplification, targeting the V1–V3 regions of the 16S rRNA gene. Library preparation was performed using the universal bacterial primer pair 27 F (AGAGTTTGATCMTGGCTCAG) and 519R (GWATTACCGCGGCKGCTG) (David 1991) followed by sequencing on the Illumina MiSeq platform using 300-bp paired-end (PE) reads. A total of 71 specimens of *L. c. dorsalis* from sheep-growing regions and eight specimens of *L. c. cuprina* from urban regions were analysed, yielding nearly 25 million paired-end reads.

### Data analysis

Paired-end reads were assembled by aligning the forward and reverse reads using PEAR1 (v. 0.9.5) (Zhang et al. 2014). Primer sequences were identified and trimmed (Trimmomatic v.0.38) before downstream processing (Bolger et al. 2014). Trimmed sequences were processed using Quantitative Insights into Microbial Ecology (QIIME 1.8) (Caporaso et al. 2010), together with USEARCH (v.8.0.1623) (Edgar 2010; Edgar et al. 2011) and the UPARSE pipeline. Sequences were quality filtered (USEARCH tools), and full-length duplicate sequences were removed and sorted by abundance. Singletons or unique reads in the data set were discarded. Sequences were clustered and then chimera filtered using “rdp_gold” database as a reference. To obtain the number of reads in each operational taxonomic unit (OTU), reads were mapped back to OTUs with a minimum identity of 97%. QIIME taxonomy was assigned using Greengenes database (v.13_8, Aug 2013) (DeSantis et al. 2006). A spreadsheet containing OTU counts for all identified genera and species per sample was generated using Microsoft Excel to facilitate further comparative analysis between the two* L. cuprina* subspecies.

Alpha diversity was calculated from the OTU count table after rarefaction to 26,000 reads per sample to minimize the influence of unequal sequencing depth among samples. Three alpha-diversity metrics were calculated: observed OTU richness, Shannon diversity (Shannon 1948), and Simpson diversity (Simpson 1949). Observed OTUs were used as a measure of bacterial richness, while Shannon and Simpson indices were used to assess diversity while accounting for both richness and evenness of bacterial taxa. Alpha-diversity metrics were calculated using the vegan package in R (v4.2.3) (Oksanen 2015; Team 2022). Differences in alpha diversity between *L. c. dorsalis* and *L. c. cuprina* were assessed using Wilcoxon rank-sum tests, as the sample sizes between subspecies were uneven and normality could not be assumed (Wilcoxon 1945). Boxplots were generated using ggplot2 with individual points overlaid to show the distribution of biological samples within each subspecies (Wickham 2016). Relative abundances were calculated separately at the phylum and genus taxonomic levels as the percentage of total abundance per sample. Differences in relative abundance between *L. c. dorsalis* and *L. c. cuprina* were assessed independently for each taxon using Wilcoxon rank-sum tests, with *p*-values adjusted for multiple comparisons using the Benjamini–Hochberg false discovery rate (FDR) correction (Benjamini and Hochberg 1995; Wilcoxon 1945). For phylum-level comparisons, dominant phyla were visualized using bar plots showing mean relative abundance ± standard error. For genus-level comparisons, the ten most dominant genera, selected based on mean relative abundance across all samples, were visualized using boxplots with individual sample points overlaid.

β-Diversity analysis was performed to assess differences in bacterial community composition between *L. c. dorsalis* and *L. c. cuprina*. Bray–Curtis dissimilarity matrices were calculated based on relative abundance data using the vegdist function in the vegan package in R (v4.2.3) (Bray and Curtis 1957; Oksanen 2015; Team 2022). Principal coordinates analysis (PCoA) was performed using the cmdscale function to visualize patterns of bacterial community similarity among samples. Statistical differences in community composition between subspecies were assessed using permutational multivariate analysis of variance (PERMANOVA) implemented with the adonis2 function using 999 permutations (Anderson 2001). To assess homogeneity of multivariate dispersion between groups, analysis of multivariate homogeneity of group dispersions (betadisper) was performed followed by analysis of variance (ANOVA) (Oksanen 2015).

Predicted functional profiling of bacterial communities was performed using PICRUSt2 (Douglas et al. 2020). Sequence placement within the PICRUSt2 pipeline was performed using EPA-ng and downstream phylogenetic placement tools (Czech et al. 2020; Douglas et al. 2020). Predicted pathway profiles were generated at the MetaCyc pathway level, and stratified outputs were retained to examine OTU-level contributions to predicted pathways (Caspi et al. 2018). The reliability of predicted functional profiles was assessed using Nearest Sequenced Taxon Index (NSTI) values (Douglas et al. 2020; Langille et al. 2013). Variation in predicted functional pathway composition among samples was assessed. MetaCyc pathway abundance profiles were converted to relative abundance and Bray–Curtis dissimilarities were calculated (Bray and Curtis 1957). PCoA was used to visualize differences in predicted functional composition among samples. PERMANOVA was performed using adonis2 in the R package vegan with 999 permutations to test for differences in predicted pathway composition between the subspecies (Oksanen 2015). Homogeneity of multivariate dispersion was assessed using betadisper and permutation tests (Oksanen 2015).

Predicted MetaCyc pathway abundance profiles were used to compare inferred microbial functional potential between *L. c. dorsalis* and *L. c. cuprina*. For each predicted pathway, mean relative abundance was calculated separately for each subspecies. Differences in predicted pathway abundance between subspecies were assessed using Wilcoxon rank-sum tests, and *p*-values were adjusted for multiple testing using the Benjamini–Hochberg FDR correction (Benjamini and Hochberg 1995; Wilcoxon 1945). Log2 fold changes were calculated as the mean relative abundance in *L. c. dorsalis* relative to *L. c. cuprina*, using a small pseudocount to avoid division by zero. To visualize variation in predicted pathway profiles among samples, the 50 most variable PICRUSt2-predicted MetaCyc pathways were displayed as a heatmap using the pheatmap package in R (v4.2.3) (Kolde 2019). Pathway abundance values were converted to relative abundance, log10 transformed using a small pseudocount, and scaled by pathway using *z*-score standardization. The 50 pathways were selected based on the highest variance in relative abundance across samples. Samples were annotated according to subspecies, and hierarchical clustering was used to examine similarities in predicted functional profiles among samples and pathways. Pathways showing the strongest differences between subspecies were visualized using a horizontal bar plot, with positive log2 fold-change values indicating higher predicted abundance in *L. c. dorsalis* and negative values indicating higher predicted abundance in *L. c. cuprina*.

## Results

### High-quality 16S rRNA sequencing data confirm distinct *Lucilia cuprina* subspecies representation

In total, 71 blowflies collected from sheep-farming regions of New South Wales, Victoria, and Western Australia were identified morphologically as *L. c. dorsalis* and eight specimens from urban regions of Queensland were identified as *L. c. cuprina* (Fig. 1, Table 1). A total of 24,688,935 paired-end reads (14.84 Gb) were obtained via 16S rRNA sequencing. All the raw sequence files were submitted to the GenBank Sequence Read Archive (SRA) under the BioProject accession number PRJNA1313656.

### *Lucilia cuprina dorsalis* and *L. c. cuprina* harbor compositionally distinct bacterial communities

**Fig. 2:**
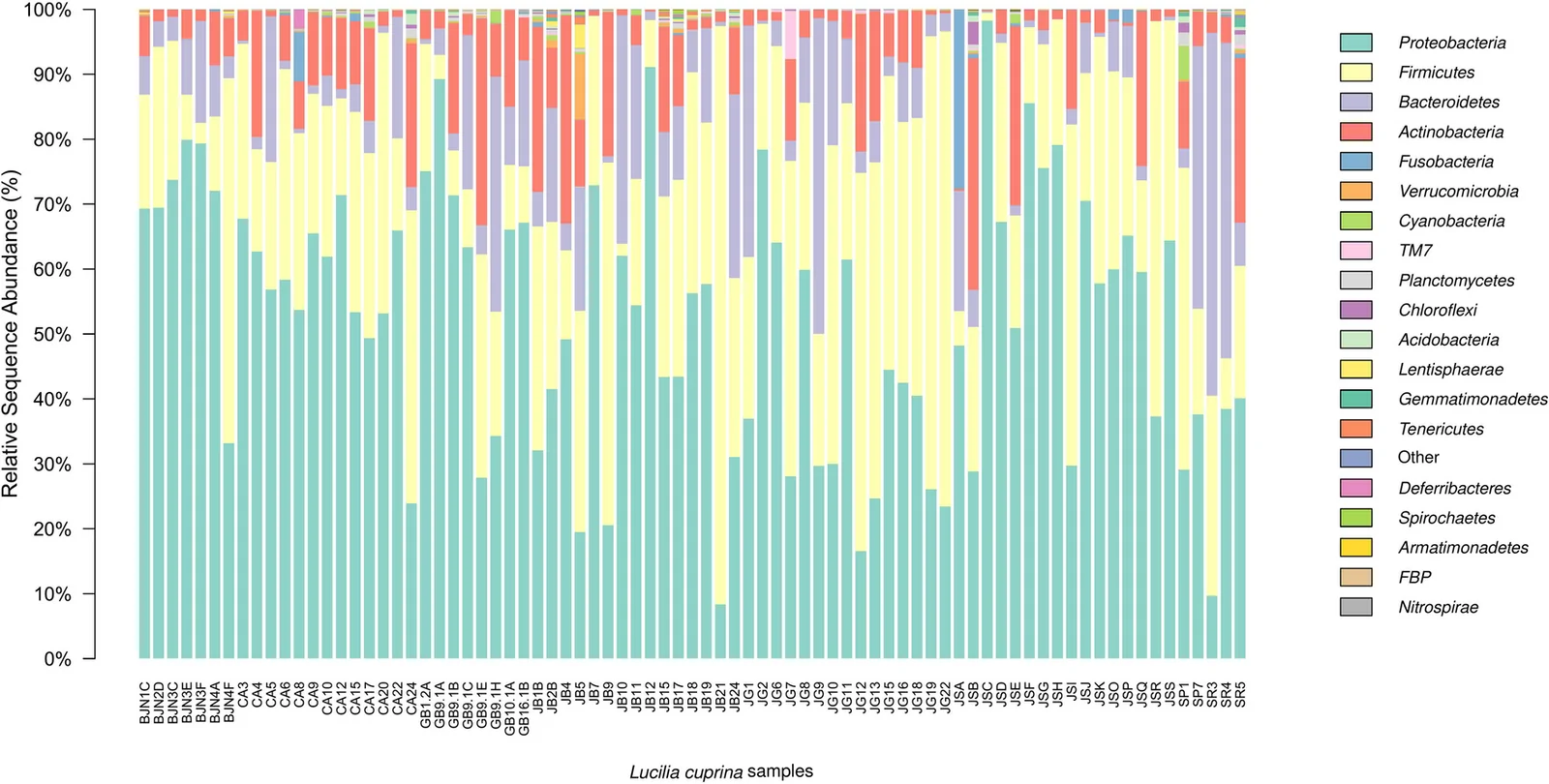
Relative abundance of dominant bacterial phyla associated with *Lucilia cuprina* subspecies based on 16S rRNA gene sequencing. Stacked bar plots represent the proportional relative abundance of the dominant bacterial phyla detected across individual samples of *Lucilia cuprina dorsalis* and *Lucilia cuprina cuprina*. Each bar represents a single sample, and colors correspond to different bacterial phyla. Low-abundance taxa were grouped into the “Other” category

**Fig. 3:**
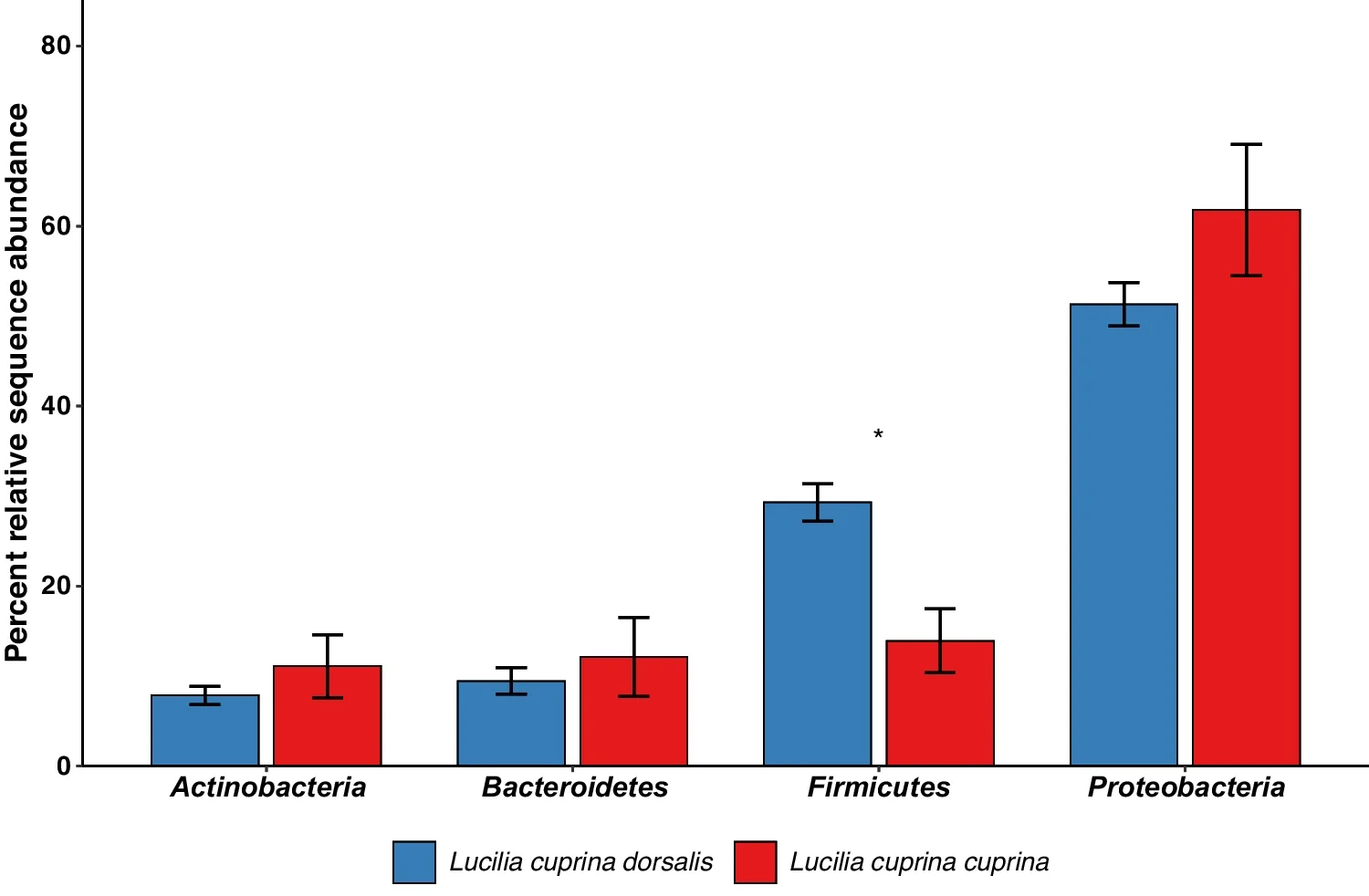
Phylum-level bacterial community composition associated with *Lucilia cuprina dorsalis* and *Lucilia cuprina cuprina*. Bar plots show the mean percent relative sequence abundance of the four dominant bacterial phyla: *Proteobacteria*, *Firmicutes*, *Bacteroidetes*, and *Actinobacteria*. Error bars represent the standard error of the mean. Differences in relative abundance between subspecies were assessed using Wilcoxon rank-sum tests, with *p*-values adjusted using the Benjamini–Hochberg false discovery rate (FDR) correction. Only significant FDR-adjusted comparisons are indicated on the plot; *Firmicutes* differed significantly between subspecies

For each *L. c. dorsalis* and *L. c. cuprina* specimen, the total bacterial abundance representing distinct phyla was established and then compared between the subspecies (Fig. 2). More than 98% of the bacterial sequences obtained from the two *Lucilia* subspecies represented the phyla *Proteobacteria*, *Firmicutes*, *Bacteroidetes*, and *Actinobacteria* (Fig. 3; Table 2); however, the microbial communities of *L. c. dorsalis* and *L. c. cuprina* were composed of distinct phylum-level profiles. In *L. c. dorsalis*, the community was composed primarily of *Proteobacteria* (51.3%), *Firmicutes* (29.3%), *Bacteroidetes* (9.5%), and *Actinobacteria* (7.9%). In contrast, *L. c. cuprina* was dominated by *Proteobacteria* (61.8%), with lower relative abundances of *Firmicutes* (13.9%), *Bacteroidetes* (12.1%), and *Actinobacteria* (11.1%). Statistical comparison of phylum-level relative abundance showed that *Firmicutes* differed significantly between the two subspecies after Benjamini–Hochberg FDR correction (Wilcoxon rank-sum test, FDR = 0.030), whereas *Actinobacteria*, *Bacteroidetes*, and *Proteobacteria* did not differ significantly after multiple-testing correction (Fig. 3). These findings indicate that, while both subspecies shared broadly similar dominant phyla, the increased relative abundance of *Firmicutes* in *L. c. dorsalis* contributed to phylum-level differences between the two bacterial communities (Supplementary Table 1).

**Table 2:** Percentage relative sequence abundance of bacteria associated with *Lucilia* subspecies at the phyla level

Phylum	*Lucilia cuprina dorsalis*	*Lucilia cuprina cuprina*
*Proteobacteria*	51.32	61.8
*Firmicutes*	29.31	13.93
*Bacteroidetes*	9.47	12.14
*Actinobacteria*	7.88	11.1
*Fusobacteria*	0.65	0.03
*Verrucomicrobia*	0.25	0.08
*Cyanobacteria*	0.21	0.31
*TM7*	0.18	0.08
*Planctomycetes*	0.15	0.17
*Chloroflexi*	0.14	0.06
*Acidobacteria*	0.11	0.13
*Lentisphaerae*	0.08	0
*Gemmatimonadetes*	0.07	0.06
*Tenericutes*	0.07	0.01
*Deferribacteres*	0.05	0
*Spirochaetes*	0.03	0
*Armatimonadetes*	0.02	0.03
*FBP*	0.02	0.02
*BRC1*	0.01	0
*Chlamydiae*	0.01	0
*Chlorobi*	0.01	0
*Elusimicrobia*	0.01	0
*Fibrobacteres*	0.01	0
*Kazan-3B-28*	0.01	0
*LD1*	0.01	0
*NC10*	0.01	0
*Nitrospirae*	0.01	0.07
*OD1*	0.01	0.01
*OP11*	0.01	0.02
*SC4*	0.01	0
*SR1*	0.01	0
*Synergistetes*	0.01	0
*TM6*	0.01	0
*WPS-2*	0.01	0.01
*WS2*	0.01	0
*Thermi*	0.01	0
*MVP-21*	0	0.01
*Unclassified*	0.01	0.01
*Classified*	99.99	99.99

The bacterial communities associated with the two *L. cuprina* subspecies also showed distinct differences at the class level, with *L. c. dorsalis* being composed primarily of *Gammaproteobacteria* (33.8%) and *Bacilli* (23.4%) and *L. c. cuprina* harboring a higher proportion of *Gammaproteobacteria* (38.2%) but a lower abundance of *Bacilli* (12.9%). Other notable classes include *Alphaproteobacteria*, which were present in similar proportions in both subspecies (i.e., *L. c. dorsalis*, 13.1%; *L. c. cuprina*, 13%), and *Actinobacteria*, which were more abundant in *L. c. cuprina* (10.8%) than in *L. c. dorsalis* (7.6%). Members of the *Flavobacteriia* class were present at comparable levels in both subspecies (i.e., *L. c. dorsalis*, 6.4%; *L. c. cuprina*, 6.7%) but distinct variations were observed within the *Clostridia*, which were more prevalent in *L. c. dorsalis* (5.5%) compared to *L. c. cuprina* (0.9%) and *Betaproteobacteria* which were more abundant in *L. c. cuprina* (11%) than in *L. c. dorsalis* (4%). Additionally, *Bacteroidia* was present at low levels in both subspecies but at higher proportions in *L. c. dorsalis* (2.7%) compared to *L. c. cuprina* (0.2%). In contrast, *Sphingobacteriia* was notably more abundant in *L. c. cuprina* (3.9%) than in *L. c. dorsalis* (0.5%) (Supplementary Table 1).

The most abundant bacterial orders for both subspecies included *Pseudomonadales* (13.9%), *Enterobacteriales* (12.5%), *Lactobacillales* (11.3%), *Actinomycetales* (9.2%), *Xanthomonadales* (8.5%), *Rhodospirillales* (8.7%), *Bacillales* (6.7%), and *Flavobacteriales* (6.5%). In *L. c. dorsalis*, *Lactobacillales* (15.5%), *Xanthomonadales* (14.6%), *Rhodospirillales* (11.5%), and *Enterobacteriales* (11.4%) were among the dominant orders, whereas in *L. c. cuprina*, *Pseudomonadales* (21.6%), *Enterobacteriales* (13.5%), *Actinomycetales* (10.8%), and *Burkholderiales* (8.1%) were more prevalent. Some bacterial orders, such as *Clostridiales* (5.4% in *L. c. dorsalis*, 0.9% *in L. c. cuprina*) and *Bacteroidales* (2.4% in *L. c. dorsalis*, 0.2% in *L. c. cuprina*), were more abundant in *L. c. dorsalis*, while *Rhizobiales* (4.9% in *L. c. cuprina*, 0.9% in *L. c. dorsalis*) and *Sphingobacteriales* (3.9% in *L. c. cuprina*, 0.5% in *L. c. dorsalis*) were mostly found in *L. c. cuprina* (Supplementary Table 1).

The bacterial family composition of *L. c. dorsalis* and *L. c. cuprina* also demonstrated differences. In *L. c. dorsalis*, dominant families included *Xanthomonadaceae* (14.5%), *Acetobacteraceae* (11.4%), and *Enterobacteriaceae* (11.4%), whereas in *L. c. cuprina*,* Enterobacteriaceae* (13.5%), *Pseudomonadaceae* (12.9%), and *Moraxellaceae* (8.7%) were more prevalent. Several bacterial families had notable differences in relative abundance between the two *L. cuprina* subspecies. For example, *Staphylococcaceae* (4.4% in *L. c. dorsalis*, 1.1% in *L. c. cuprina*) and *Streptococcaceae* (3.9% in *L. c. dorsalis*, 0.3% in *L. c. cuprina*) were more prevalent in *L. c. dorsalis*. Conversely, *Comamonadaceae* (0.2% in *L. c. dorsalis*, 5.8% in *L. c. cuprina*) and *Sphingobacteriaceae* (0.5% in *L. c. dorsalis*, 3.9% in *L. c. cuprina*) were more abundant in *L. c. cuprina* (Supplementary Table 1)*.*

**Fig. 4:**
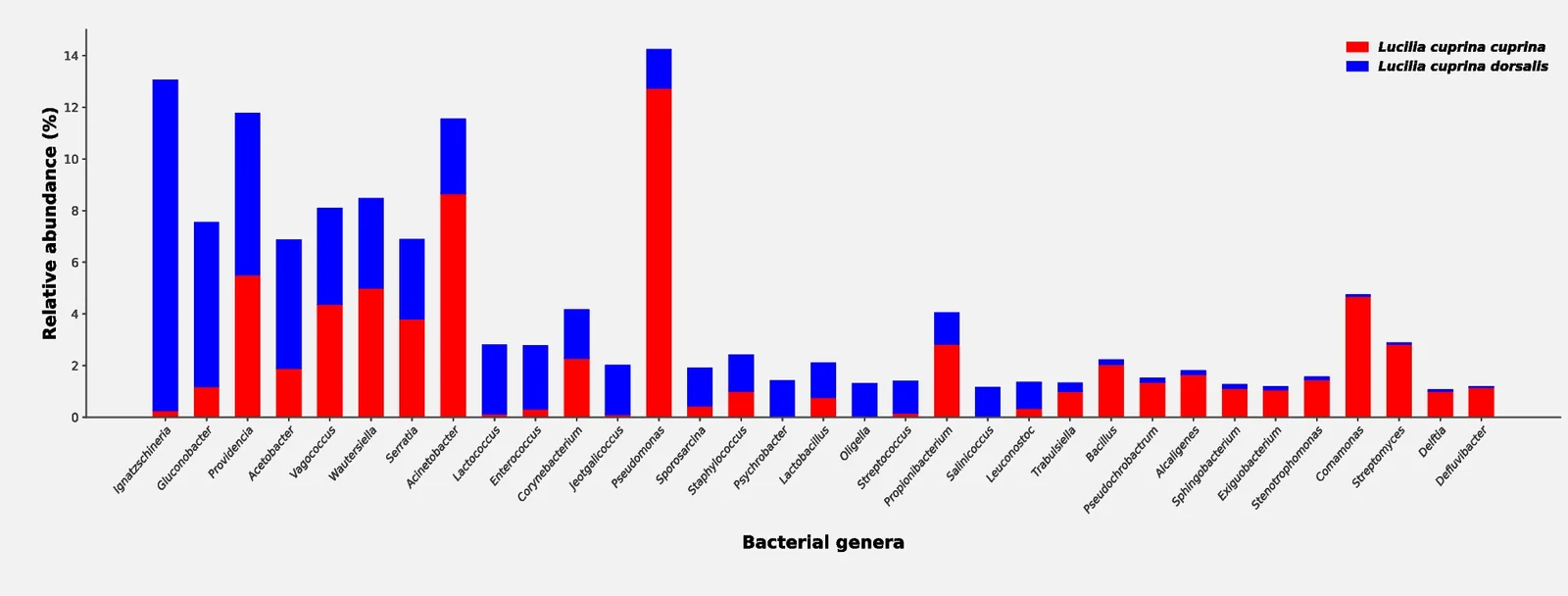
Relative abundance of dominant bacterial genera associated with *Lucilia cuprina* subspecies based on 16S rRNA gene sequencing. Bar plots show the relative abundance (%) of bacterial genera detected at ≥ 1% abundance across *Lucilia cuprina dorsalis* and *Lucilia cuprina cuprina* samples. Colors indicate subspecies, with blue representing *Lucilia cuprina dorsalis* and red representing *Lucilia cuprina cuprina*. Genera are shown on the *x*-axis and relative abundance is shown on the *y*-axis

**Fig. 5:**
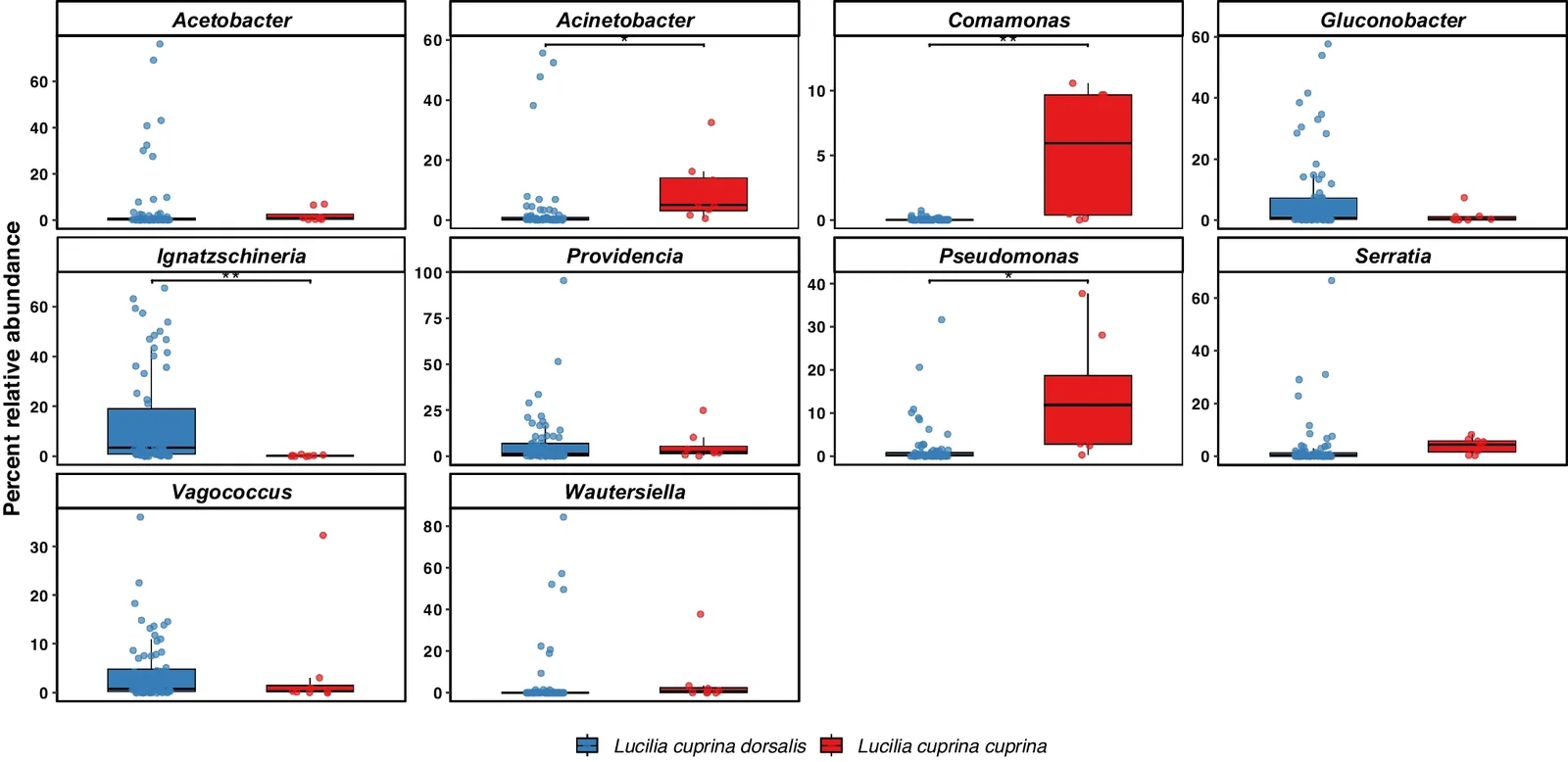
Subspecies-specific variation in dominant bacterial genera associated with *Lucilia cuprina*. Boxplots show the percent relative sequence abundance of the dominant bacterial genera across individual samples. Boxes represent the median and interquartile range, and points represent individual samples. Blue indicates *Lucilia cuprina dorsalis* and red indicates *Lucilia cuprina cuprina*. Differences between subspecies were assessed using Wilcoxon rank-sum tests, with *p*-values adjusted across all tested genera using the Benjamini–Hochberg false discovery rate (FDR) correction. Statistical annotations are shown only for genera with significant FDR-adjusted differences and genera without annotations were not significant after FDR correction

A comparative analysis of genus distribution across the two *L. cuprina* subspecies revealed *Pseudomonas* as the predominant genus, displaying the highest relative abundance across all profiles (14.2%). This was followed by *Ignatzschineria* (13.1%), *Providencia* (11.8%), and *Acinetobacter* (11.5%), all of which were major contributors to the bacterial communities associated with *L. c. dorsalis* and *L. c. cuprina* (Fig. 4; Supplementary Table 1). To further assess genus-level differences between subspecies, relative abundances were compared using Wilcoxon rank-sum tests with Benjamini–Hochberg FDR correction. This analysis showed significant subspecies-associated shifts in several bacterial genera, with *Ignatzschineria* (12.8%) enriched in *L. c. dorsalis*, whereas *Pseudomonas* (12.7%), *Comamonas* (4.7%), and *Acinetobacter* (8.7%) were more abundant in *L. c. cuprina* (Fig. 5; Supplementary Table 2). These statistically supported genus-level differences indicate that the bacterial communities associated with *L. c. dorsalis* and *L. c. cuprina* differ in the relative abundance of key dominant taxa.

**Fig. 6:**
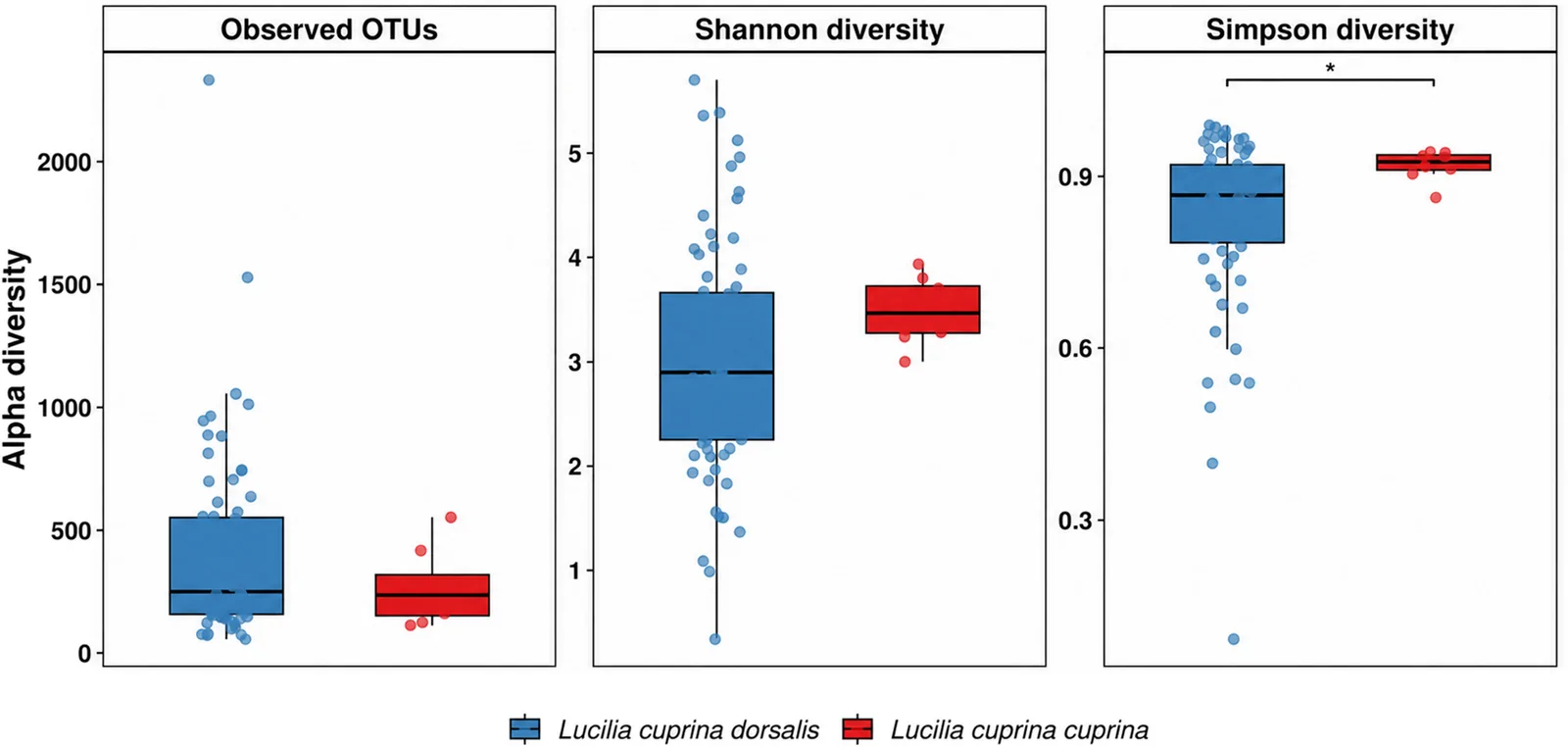
Alpha diversity of bacterial communities associated with *Lucilia cuprina* subspecies. Boxplots show observed OTU richness, Shannon diversity, and Simpson diversity calculated from the OTU table after rarefaction to 26,000 reads per sample. Each point represents an individual sample. Boxes represent the interquartile range, horizontal lines represent medians, and whiskers show the distributional spread of the data. Blue indicates *Lucilia cuprina dorsalis* and red indicates *Lucilia cuprina cuprina*. Differences between subspecies were assessed using Wilcoxon rank-sum tests. Statistical annotations are shown only for significant comparisons; Simpson diversity differed significantly between subspecies whereas observed OTU richness and Shannon diversity were not significant

Alpha-diversity analysis was performed to compare bacterial richness and diversity between *L. c. dorsalis* and *L. c. cuprina*. Observed OTU richness did not differ significantly between the two subspecies, indicating that the overall number of detected bacterial OTUs was broadly comparable between groups after rarefaction to 26,000 reads per sample (Wilcoxon rank-sum test, *p* = 0.455); Shannon diversity did not differ significantly between the subspecies (*p* = 0.125), suggesting that overall bacterial community diversity was not strongly differentiated between the two groups. In contrast, Simpson diversity showed a modest significant difference between the *L. cuprina* subspecies (*p* = 0.045) (Fig. 6).

**Fig. 7:**
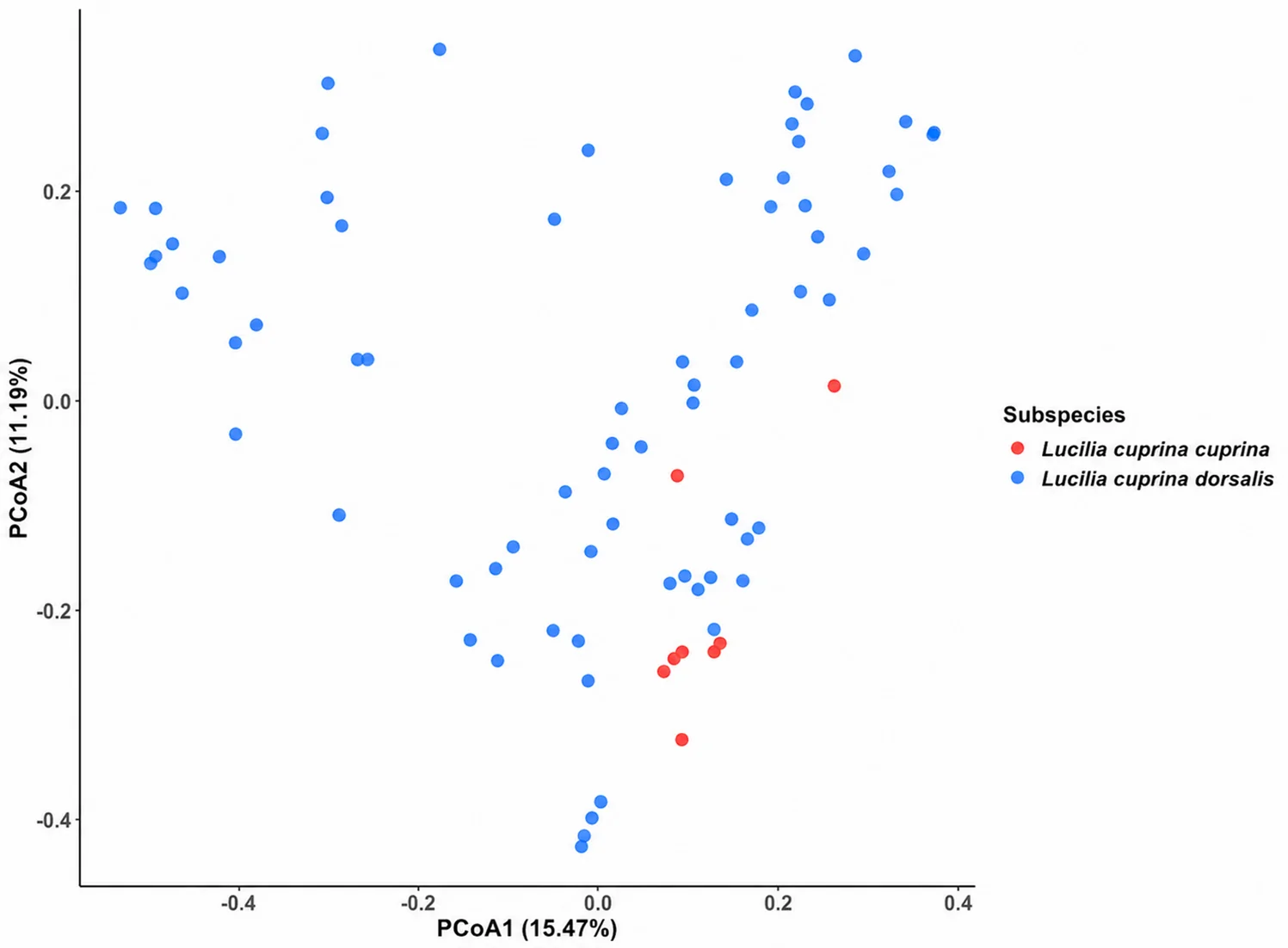
Principal coordinates analysis (PCoA) of bacterial community composition in *Lucilia cuprina* subspecies based on Bray–Curtis dissimilarity. Each point represents an individual sample, with colours indicating subspecies: *Lucilia cuprina dorsalis* in blue *and Lucilia cuprina cuprina* in red. The first two PCoA axes explained 15.47% and 11.19% of the total variation, respectively

The β-diversity analysis based on Bray–Curtis dissimilarity was used to determine whether bacterial community composition differed between *L. c. dorsalis* and *L. c. cuprina*. PCoA showed separation between samples from the two subspecies, indicating differences in overall bacterial community structure (Fig. 7). Although some overlap among samples was observed, the ordination suggested that the bacterial communities associated with *L. c. dorsalis* and *L. c. cuprina* were compositionally distinct. PERMANOVA based on Bray–Curtis dissimilarity confirmed a significant difference in bacterial community composition between the two subspecies (*R*^2^ = 0.046, *F* = 3.693, *p* = 0.001). This result indicates that subspecies identity was significantly associated with variation in bacterial community structure.

### *Lucilia cuprina dorsalis* from sheep-growing regions exhibit microbiomes enriched in *Ignatzschineria*, *Gluconobacter*, and lactic acid bacteria

**Fig. 8:**
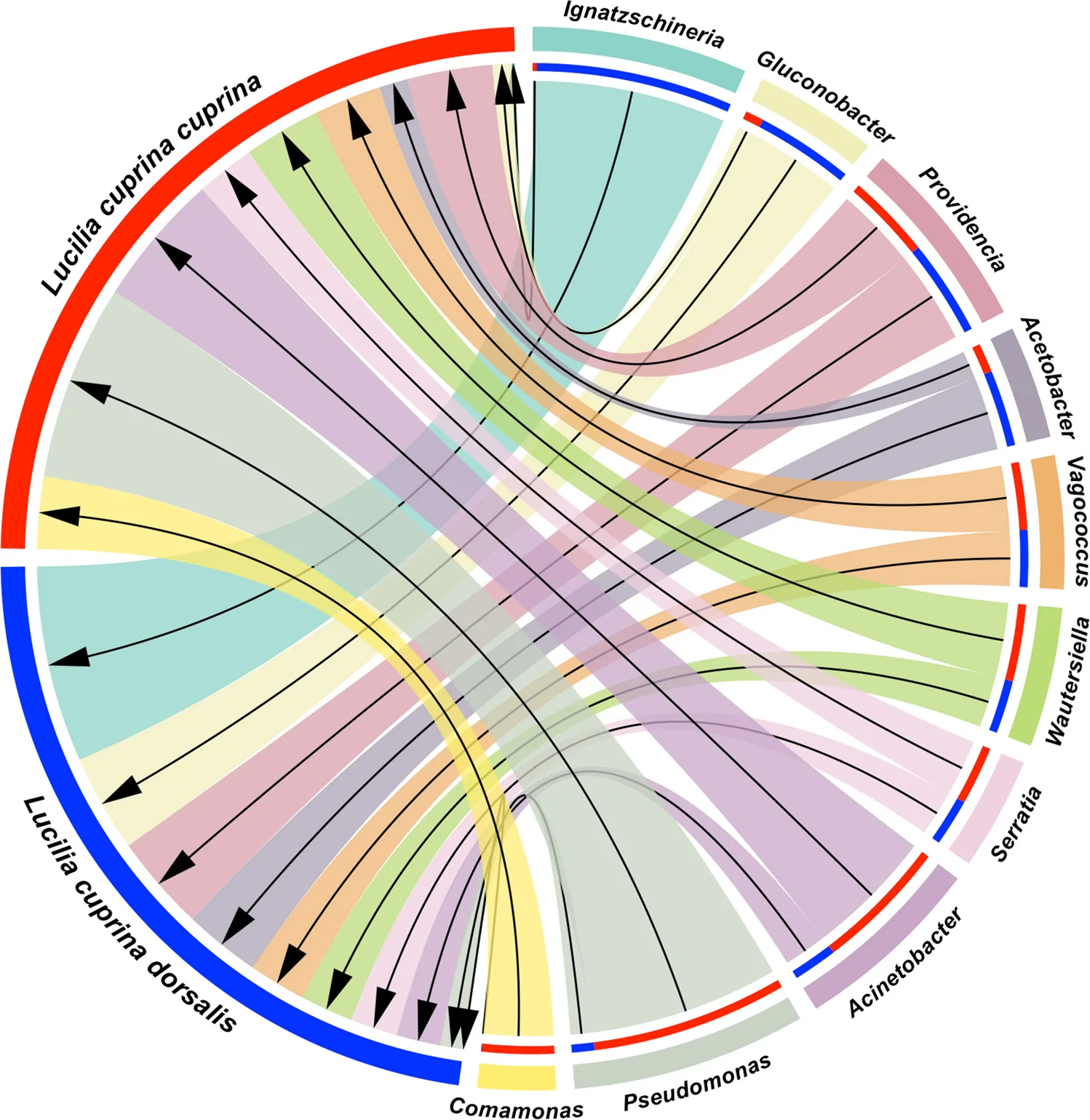
Chord diagram showing the relative association and distribution of the dominant bacterial genera between *Lucilia cuprina dorsalis* (blue arc) and *Lucilia cuprina cuprina* (red arc). The width of each chord corresponds to the relative abundance contribution of individual bacterial genera within each subspecies. Colored outer segments represent individual bacterial genera while connecting ribbons depict the proportional contribution of each genus to one or both subspecies. Directional arrows indicate the flow from bacterial genera to host subspecies. Genera shared between subspecies are connected to both arcs, while unique genera are linked to only one

Microbiome analysis of *L. c. dorsalis* samples from sheep-growing regions in Australia identified diverse and compositionally rich bacterial communities. The predominant bacterial genera identified belonged to the phyla *Proteobacteria*, *Firmicutes*, *Bacteroidetes*, and *Actinobacteria*, with the most abundant genus being *Ignatzschineria* (*Gammaproteobacteria*; *Xanthomonadaceae*), comprising 12.8% of the bacterial community. Other *Proteobacteria* included *Gluconobacter* (6.3%), *Providencia* (6.2%), *Acetobacter* (5%), and *Serratia* (3.1%) (Fig. 5). Among the *Firmicutes*, the predominant genera included *Vagococcus* (3.7%), *Lactococcus* (2.7%), *Enterococcus* (2.4%), and *Staphylococcus* (1.4%). Within *Bacteroidetes*, *Wautersiella* (3.5%) was also identified, while *Actinobacteria* were represented by genera such as *Corynebacterium* (1.9%) and *Propionibacterium* (1.2%) (Fig. 8; Supplementary Table 1).

### Urban *Lucilia cuprina cuprina* display microbiomes dominated by *Pseudomonas*, *Acinetobacter*, and *Comamonas*

The microbiome analysis of *L. c. cuprina* from urban regions in Australia revealed a diverse bacterial community, primarily dominated by *Proteobacteria*, *Firmicutes*, *Actinobacteria*, and *Bacteroidetes.* The *Proteobacteria* constituted a significant portion, with *Pseudomonas* (12.7%) as the most abundant genus, followed by *Acinetobacter* (8.7%), *Providencia* (5.5%), *Serratia* (3.8%), and *Comamonas* (4.7%) (Fig. 5). The *Firmicutes* were represented by *Vagococcus* (4.8%), *Bacillus* (2.0%), *Exiguobacterium* (1.1%), and *Staphylococcus* (1.0%). Among the *Actinobacteria*,* Propionibacterium* (2.8%), *Streptomyces* (2.8%), and *Corynebacterium* (2.3%) were present and members of *Bacteroidetes* included *Wautersiella* (5%) and *Sphingobacterium* (1.1%), highlighting the microbial diversity associated with this subspecies (Fig. 8; Supplementary Table 1).

### Subspecies differ in genus-level diversity with distinct and shared core microbiota

**Fig. 9:**
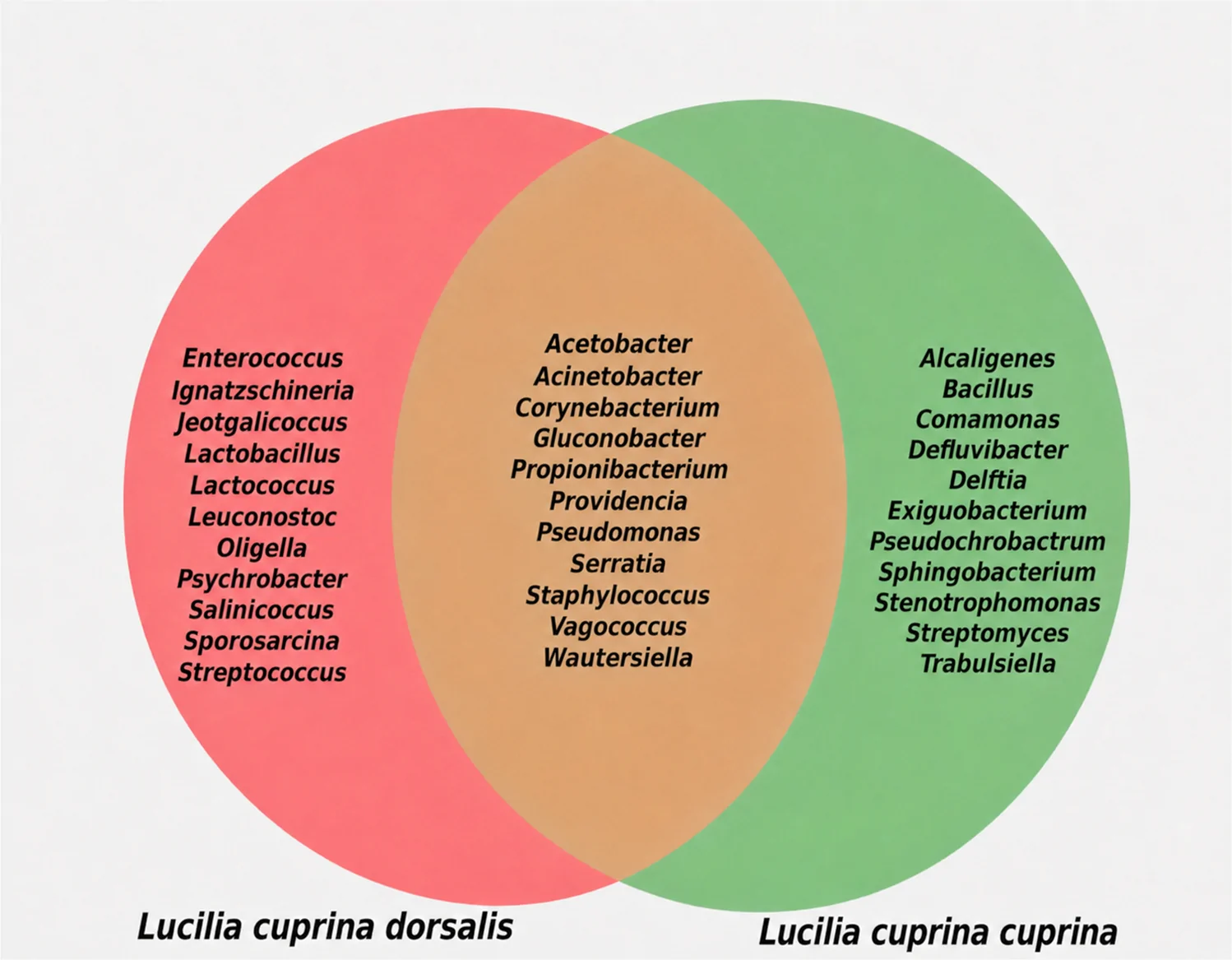
Venn diagram showing bacterial genera (≥ 1%) associated with the two *Lucilia cuprina* subspecies. A total of 11 bacterial genera were shared between the subspecies

The bacterial composition of the two *L. cuprina* subspecies differed markedly, reflecting variations in microbial diversity and abundance. *L. c. dorsalis* contained 11 unique genera, including *Ignatzschineria* (12.8%), *Lactococcus* (2.7%), *Enterococcus* (2.4%), *Jeotgalicoccus* (1.9%), *Sporosarcina* (1.4%)*, Psychrobacter* (1.4%), *Lactobacillus* (1.3%), *Oligella* (1.3%), *Streptococcus* (1.2%), *Salinicoccus* (1.2%), and *Leuconostoc* (1.0%). In contrast, *L. c. cuprina* contained 11 unique genera including *Comamonas* (4.7%), *Streptomyces* (2.8%), *Bacillus* (2.0%)*, Alcaligenes* (1.7%), *Stenotrophomonas* (1.5%), *Pseudochrobactrum* (1.4%), *Defluvibacter* (1.2%), *Sphingobacterium* (1.1%), *Exiguobacterium* (1.1%), *Delftia* (1.0%), and *Trabulsiella* (1.0%) (Fig. 6). Despite these differences, 11 genera—*Acetobacter*, *Pseudomonas*, *Providencia*, *Propionibacterium*, *Acinetobacter*, *Vagococcus*, *Wautersiella*, *Staphylococcus*, *Gluconobacter*, *Serratia*, and *Corynebacterium—*were common to both subspecies, though their relative abundances varied (Fig. 9).

### Subspecies associated differences in predicted microbial metabolic pathways

PICRUSt2 was used to infer the predicted functional potential of bacterial communities associated with *L. cuprina* subspecies samples. The reliability of predictions was supported by low abundance-weighted NSTI values across samples, with a mean of 0.099 and a median of 0.091, indicating that dominant OTUs were generally well represented in available reference genome databases. PCoA based on Bray–Curtis dissimilarities of predicted MetaCyc pathway abundances showed partial separation between *L. c. cuprina* and *L. c. dorsalis* samples. The first two PCoA axes explained 26.3% and 17.5% of the variation, respectively, accounting for 43.8% of the total variation in predicted pathway composition. The *L. c. dorsalis* samples showed greater dispersion across the ordination space, whereas *L. c. cuprina* samples appeared more tightly clustered (Supplementary Fig. 1). PERMANOVA indicated a significant difference in predicted functional pathway composition between subspecies, explaining 5.5% of the variation in pathway profiles (*R*^2^ = 0.055, *F* = 4.503, *p* = 0.003).

Predicted MetaCyc pathway profiles were generated and visualized using a heatmap of the 50 most variable pathways across samples. The heatmap showed variation in predicted pathway abundance across individual samples, with several pathways displaying higher relative abundance in subsets of either *L. c. dorsalis* or *L. c. cuprina* (Supplementary Fig. 2). Hierarchical clustering indicated partial grouping of samples according to predicted functional profiles, although complete separation between subspecies was not observed. This suggests that the bacterial communities associated with the two subspecies share many predicted functional pathways, while also differing in the relative abundance of selected metabolic functions. Several of the highly variable predicted pathways were associated with lipid metabolism, nucleotide metabolism, amino acid biosynthesis, carbohydrate degradation, energy metabolism, and cell-wall related processes. Pathways such as fatty acid biosynthesis, palmitate biosynthesis, stearate biosynthesis, TCA cycle-associated pathways, nucleotide salvage and degradation pathways, and peptidoglycan biosynthesis contributed strongly to the observed functional variation.

Differential analysis of the predicted MetaCyc pathways identified several pathways with differing predicted relative abundance between *L. c. dorsalis* and *L. c. cuprina* (Supplementary Fig. 3, Supplementary Table 3). Pathways with higher predicted abundance in *L. c. dorsalis* included peptidoglycan biosynthesis pathways, coenzyme A biosynthesis, inosine-5′-phosphate biosynthesis, adenosine and purine nucleotide biosynthesis, aromatic amino acid biosynthesis, and the Calvin-Benson-Bassham cycle. These pathways suggest increased predicted representation of microbial biosynthetic functions in the *L. c. dorsalis*–associated bacterial community including pathways involved in bacterial cell-wall biosynthesis. In contrast, pathways with higher predicted abundance in *L. c. cuprina* included l-arginine degradation, methylglyoxal degradation, creatinine degradation, syringate degradation, methylgallate degradation, gallate degradation, vanillin and vanillate degradation, and protocatechuate degradation. These pathways indicate higher predicted representation of amino acid, nitrogenous-compound, and aromatic-compound degradation functions in the *L. c. cuprina*–associated bacterial community (Supplementary Fig. 3).

## Discussion

The results of this study provide significant insights into the microbiomes of two closely related *Lucilia* subspecies: *L. c. dorsalis*, which inhabits sheep-growing regions and is predominantly parasitic, and the necrophagous *L. c. cuprina*, which is found in urban regions of Australia (Anstead et al. 2016; Kapoor et al. 2023; Nelson et al. 2012). The present study focused specifically on head-associated bacterial communities, including microbes potentially associated with feeding structures and salivary tissues.

The bacterial communities associated with the two subspecies of *L. cuprina* showed notable differences in their microbial composition and relative abundances indicating subspecies associated variation in community structure, potentially reflecting differences in ecology, habitat, or host physiology. At the phylum level, both subspecies were predominantly composed of *Proteobacteria* and *Firmicutes*, *Bacteroidetes*, and *Actinobacteria*, as seen in previous studies of *M. domestica* (Gupta et al. 2012), *Bactrocera carambolae* and *B. dorsalis* (Yong et al. 2017), *Sarcophaga* spp. (Gupta et al. 2014), and blowflies *L. sericata* and *L. cuprina* (Gasz et al. 2021; Greeff et al. 2021; Iancu et al. 2021; Junqueira et al. 2017; Palevich et al. 2021; Singh et al. 2015; Tomberlin et al. 2017). Collectively, these phyla accounted for over 98% of the bacterial sequences. However, the proportional representation of these phyla differed markedly between the subspecies with *L. c. dorsalis* exhibiting a more balanced community composition whereas *L. c. cuprina* was characterized by a strong predominance of *Proteobacteria* (61.8%), with the remaining phyla contributing smaller fractions (i.e., *Firmicutes* (13.9%), *Bacteroidetes* (12.1%), and *Actinobacteria* (11.1%)) of the total community. Among the dominant phyla, *Firmicutes* was the only phylum that differed significantly between the two subspecies after Benjamini–Hochberg FDR correction, being more abundant in *L. c. dorsalis* than in *L. c. cuprina*. This suggests that the phylum-level difference between the two subspecies was driven primarily by variation in *Firmicutes* abundance rather than by broad shifts across all dominant phyla. Although *Proteobacteria* and *Firmicutes* are typically dominant members of dipteran microbiomes, *Bacteroidetes* and *Actinobacteria* are also consistently represented across several fly species (Ben‐Yosef et al. 2017; Gasz et al. 2021; Palevich et al. 2021; Singh et al. 2015; Wei et al. 2013). *Proteobacteria* are typically involved in the decomposition of organic matter (Zavarzin et al. 1991) and may play a crucial role in facilitating the blowfly’s contribution to carrion decomposition. *Firmicutes* are prevalent in soil and animal microbiota and their ability to produce endospores enables them to survive in harsh environments (Montoya et al. 2001; Sharmin et al. 2013). They are also involved in food fermentation processes (Flint et al. 2012; Lim et al. 2014). *Bacteroidetes* are major contributors to the degradation of complex carbohydrates and polysaccharides (McKee et al. 2021) and *Actinobacteria* are involved in antibiotic production, organic matter decomposition, and gut health across a range of hosts, including humans (Berdy 2005; Binda et al. 2018; Ventura et al. 2007).

The genus-level analysis provided finer resolution of bacterial community differences between *L. c. dorsalis* and *L. c. cuprina* than the phylum-level comparison. Although both subspecies shared several dominant bacterial genera, the relative abundance of key taxa differed significantly between groups after Benjamini–Hochberg FDR correction. The higher abundance of *Ignatzschineria* (12.8%) in *L. c. dorsalis* is notable because this genus has frequently been reported from necrotic, wound-associated, and fly-associated environments, suggesting that it may represent an important member of the microbiota associated with parasitic or carrion-associated dipterans. In contrast, *Pseudomonas* (12.7%), *Comamonas* (4.7%), and *Acinetobacter* (8.7%) were significantly more abundant in *L. c. cuprina*, indicating that the bacterial communities associated with this subspecies may include a greater contribution from environmentally widespread *Proteobacteria.*

*Pseudomonas* species are involved in the breakdown of organic matter and are commonly found in carrion, playing a role in nutrient cycling and decomposition (Hyde et al. 2017; Jordan et al. 2015). *Pseudomonas aeruginosa* and *P. straminea* produce pyocyanin, a compound that has been shown to attract blowflies that detect these compounds for oviposition (Emmens and Murray 1983; Gasz et al. 2021; Jordan et al. 2015; Norris et al. 2008). These bacterial species have an established role in fleece rot and the production of pyocyanin contributes to the characteristic green coloration of affected fleece (Emmens and Murray 1983). One might therefore expect *L. c. dorsalis* to have a higher abundance of *Pseudomonas* spp. within its bacterial composition given its parasitic lifestyle; however, results from this study could be influenced by when the blowflies were collected (i.e., February–April) which is not peak flystrike season (i.e., October–December and March–May) and therefore the flies are less likely to have been exposed to this bacterium during that period. *Pseudomonas aeruginosa* exhibits a remarkable capacity to acquire and disseminate plasmids, particularly those carrying antibiotic resistance and virulence genes, underscoring its significance as a versatile and opportunistic pathogen (Rocha et al. 2019; Schroeder et al. 2017). *Ignatzschineria* is also frequently associated with decomposing animal remains and is hypothesized to play a role in the degradation of organic matter (Hyde et al. 2017). It has been detected in both larvae and adult *Lucilia* spp., potentially influencing host behavior and attraction to carrion or wounds (Heddema et al. 2016; Iancu et al. 2020). Additionally, species such as *I. indica* and *I. ureiclastica* have been implicated in human wound infections, often in cases linked to blowfly maggot infestations (myiasis) (Cipolla et al. 2018; Gupta et al. 2011; Tanida et al. 2019). Other genera including *Gluconobacter*, *Providencia*, *Acetobacter*, *Vagococcus*, *Acinetobacter*, and *Wautersiella* were also prevalent in both subspecies and have been previously reported in blowflies (Gasz et al. 2021; Singh et al. 2015). These findings suggest that although both subspecies share a core set of bacterial genera, the unique contributions and relative abundances of specific genera likely reflect adaptations to different ecological niches or host interactions.

The alpha diversity analysis suggests that bacterial richness and overall diversity were broadly similar between *L. c. dorsalis* and *L. c. cuprina*. This suggests that, despite differences in ecology, habitat association, and geographic origin, both subspecies may support bacterial communities of comparable richness. The β-diversity analysis provided evidence that bacterial community composition differed between *L. c. dorsalis* and *L. c. cuprina*. PCoA based on Bray–Curtis dissimilarity showed partial separation between the two subspecies, indicating differences in overall microbial community structure. This pattern was supported by PERMANOVA, which identified a significant difference in bacterial community composition between the two groups (*R*^2^ = 0.046, *F* = 3.693, *p* = 0.001). Together, these findings indicate significant but partial differentiation in bacterial community composition between the two subspecies. The observed pattern should be interpreted as evidence of significant but moderate community-level differentiation, rather than complete separation of microbiomes between the two subspecies. These findings complement the taxonomic composition results and support the broader conclusion that parasitic and necrophagous *Lucilia* subspecies harbor distinct but overlapping microbial communities, likely shaped by a combination of host biology, ecological niche, and environmental exposure. The differences in microbial composition between rural and urban populations of *L. cuprina* indicate that ecological factors play a significant role in shaping the symbiotic relationships between these flies and their microbial communities. The subspecies identity was inherently associated with distinct geographic distributions and ecological habitats in the present study; therefore, the observed microbiome differences likely reflect a combination of host subspecies effects, environmental exposure, ecological niche specialization, and geographic variation. Future studies incorporating broader geographic sampling, sympatric population comparisons, and controlled environmental designs will be important to disentangle the relative contributions of these factors.

Our findings on the microbiome of *L. c. dorsalis* from sheep-farming regions in Australia highlight a diverse bacterial community, with key taxa potentially influencing blowfly ecology and behavior. Among the predominant bacterial phyla,* Proteobacteria*, *Firmicutes*, *Bacteroidetes*, and *Actinobacteria* were well represented, with *Ignatzschineria* emerging as the most abundant genus. Members of *Ignatzschineria* have strong chitinase activity which has been suggested to have a potential role in insect metamorphosis, likely through the degradation of chitinous structures (Tóth et al. 2001). *Ignatzschineria* spp. were previously isolated from first- and second-instar larvae of the obligate parasitic fly *Wohlfahrtia magnifica*, a major myiasis-causing species in Eurasia that significantly impacts domestic animals (Toth et al. 2006). Members of this genus have also been linked to human infections (Cipolla et al. 2018; Tanida et al. 2019) and myiasis, or myiasis-causing flies (Gupta et al. 2011; Maurin et al. 2007; Toth et al. 2006; Tóth et al. 2001). Further investigation is needed to assess the significance of their presence in *Lucilia* species and subspecies. The second most abundant genus, *Gluconobacter*, influences the microbial community structure through acid production, which can affect pH and microbial interactions. Additionally, *Gluconobacter* contributes to sugar oxidation, potentially aiding in energy production (Deppenmeier et al. 2002; Gupta et al. 2001). Another genus of note, *Providencia*, can be opportunistic or symbiotic bacteria living in soil, water, sewage, and food (Ferraresso et al. 2020; Gessew et al. 2022; Paiva et al. 2017; Shima et al. 2016). Some *Providencia* strains are pathogenic to insects, particularly *P. alcalifaciens* and *P. sneebia* in *Drosophila melanogaster* (Galac and Lazzaro 2011) and *P. alcalifaciens* and *P. rustigianii* in the Mexican fruit fly *Anastrepha ludens* (Salas et al. 2023). The bacterial diversity observed in *L. c. dorsalis* aligns with previous studies on blowfly microbiomes, highlighting the presence of both environmentally acquired and host-associated bacterial taxa (Gasz et al. 2021; Iancu et al. 2021; Palevich et al. 2021; Singh et al. 2015).

In contrast, *Pseudomonas* and *Acinetobacter* were the most abundant genera identified in *L. c. cuprina* specimens collected from urban regions of Australia. These bacteria are found in high abundance in insects associated with unsanitary environments, particularly in saprophagous and necrophagous insects (Gupta et al. 2014, 2012; Singh et al. 2015; Vogel et al. 2017; Wang et al. 2018). They are known to have a wide range of metabolic capabilities and environmental adaptability, which could be crucial for flies in urban environments feeding on diverse organic material (Singh et al. 2015). These bacteria may also help flies survive in varied environments, as they are involved in organic matter decomposition and resistance to harsh conditions (Singh et al. 2015).

Despite their microbiome differences, both *Lucilia* subspecies harbored a common group of bacterial genera (*Acetobacter*, *Pseudomonas*, *Providencia*, *Propionibacterium*, *Acinetobacter*, *Vagococcus*, *Wautersiella*, *Staphylococcus*, *Gluconobacter*, *Serratia*, and *Corynebacterium*), but their relative abundances differed markedly. Of note are several genera that may produce antiviral and antiparasitic compounds. For example, the genus *Vagococcus* has been isolated from blowflies (Singh et al. 2015), house flies (Gupta et al. 2012), fruit flies (Chandler et al. 2011), and mosquitos (Dennison et al. 2014), and are believed to produce antiviral metabolites, particularly in *Aedes albopictus* (Dennison et al. 2014). Some species or strains of *Acinetobacter* associated with the *Cephaloleia* beetles may play a role in detoxifying defensive plant compounds (Blankenchip et al. 2018). *Serratia* has been found to have a dual role in blowflies, where it is essential for some life processes (e.g., digestion), but specific strains (e.g. *Serratia marcescens*) can act as opportunistic pathogens that cause disease or death under stressful conditions or high exposure (O'callaghan et al. 1996).

The comparative analysis of the bacterial communities associated with the two sister subspecies of *L. cuprina *revealed differences in microbiome composition between specimens collected from sheep-farming and urban regions. While both subspecies share a core set of dominant taxa, the relative abundance of key bacterial groups differs substantially. Notably, *L. c. dorsalis* exhibited a higher prevalence of genera such as *Ignatzschineria* and *Gluconobacter*, whereas *L. c. cuprina* was characterized by a dominance of *Pseudomonas* and *Acinetobacter*. These distinctions suggest that variations in environmental exposure, host diet, and microhabitat conditions are likely influencing the microbial community structures, with potential implications for host physiology and pathogen dynamics. Future studies that integrate functional analyses of these microbial communities will be critical for understanding the mechanistic underpinnings of host-microbe interactions and for developing targeted strategies to manage blowfly infestations in both agricultural and urban settings.

The PICRUSt2 analysis provided an exploratory assessment of the predicted functional potential of bacterial communities associated with the parasitic and necrophagous *L. cuprina* subspecies. Predicted MetaCyc pathway profiles differed significantly between groups, suggesting that bacterial communities associated with *L. c. dorsalis* and *L. c. cuprina* may differ not only taxonomically but also in their inferred metabolic potential. This may reflect differences in ecological niche, host-associated substrates, environmental exposure, or geographic origin. Pathways predicted to be higher in *L. c. dorsalis* were mainly associated with biosynthetic processes, including peptidoglycan biosynthesis, nucleotide biosynthesis, coenzyme A biosynthesis, and aromatic amino acid biosynthesis. These predicted functions may therefore reflect differences in the dominant bacterial taxa and their biosynthetic capacities, including bacterial growth-related metabolism and cell-envelope formation. Such patterns are broadly consistent with the essential role of peptidoglycan in bacterial cell-wall structure and with evidence that insect-associated bacteria can possess metabolic functions relevant to host-associated ecological interactions (Moran et al. 2008; Vollmer et al. 2008).

In contrast, *L. c. cuprina* showed higher predicted abundance of several degradation pathways, including gallate, methylgallate, syringate, vanillin, vanillate, protocatechuate, creatinine, and l-arginine degradation. These pathways may reflect microbial capacities for degrading aromatic compounds and other organic substrates, including nitrogen-containing metabolites (Carmona et al. 2009; Díaz et al. 2013; Harwood and Parales 1996). Such predicted functions are broadly consistent with bacterial adaptation to decomposing organic matter or carrion-influenced environments, which may be relevant to necrophagous-associated microbial communities (Hyde et al. 2017; Metcalf et al. 2016; Pechal et al. 2014).

Although *Ignatzschineria* and *Pseudomonas* differed in relative abundance between *L. c. dorsalis* and *L. c. cuprina*, their functional roles cannot be inferred from 16S rRNA gene data alone. Therefore, these bacteria should be considered candidate taxa of ecological interest rather than confirmed drivers of parasitism, necrophagy, host attraction, or environmental adaptation. The observed microbial differences may reflect contrasting environmental exposures, feeding substrates, host associations, and ecological niches of the two subspecies. In addition, the unequal sample sizes between *L. c. dorsalis* and *L. c. cuprina* may have influenced the statistical power and robustness of the diversity and differential abundance analyses. Therefore, the observed microbiome differences should be interpreted with appropriate caution and validated in future studies incorporating larger and more balanced sampling designs.

Insect microbiomes can vary substantially across anatomical compartments, and the bacterial composition identified from head tissues may not fully reflect gut-associated or whole-body microbial communities. Future studies incorporating tissue-specific and whole-body microbiome comparisons, larger and more balanced sample sets, broader geographic and temporal sampling, ASV-based profiling, metagenomics, metatranscriptomics, metabolomics, culture-based approaches, and controlled microbiome manipulation experiments will be required to determine whether these bacterial taxa contribute functionally to blowfly ecology, host interactions, fitness, disease resistance, or behavior. Such integrative approaches will help clarify the biological significance of these microbial communities and may inform future management strategies for blowfly-associated challenges in agricultural and urban settings.

## Supplementary Information

Below is the link to the electronic supplementary material.

ESM 1 (XLSX 20.7 KB)

ESM 2 (CSV 98.0 KB)

ESM 3 (XLSX 52.0 KB)

ESM 4 (PNG 946 KB)

ESM 5 (PNG 1.22 MB)

ESM 6 (PNG 1.29 MB)

## Data Availability

The datasets supporting the conclusions of this article are included within the article and its Additional files. Any additional data and custom scripts used for the microbiome analysis are available from the corresponding author upon reasonable request. The datasets generated and/or analysed during the current study are publicly available in the NCBI Short Read Archive (SRA) under the accession number PRJNA1313656.
